# Shear Bond Strength of Ceramic Brackets on Enamel Conditioned With CO2 Laser

**DOI:** 10.7759/cureus.72761

**Published:** 2024-10-31

**Authors:** J. Arturo Colín-Ocampo, Rogelio J Scougall-Vilchis, Laura E Rodríguez-Vilchis, Carlo E Medina-Solís

**Affiliations:** 1 Advanced Studies and Research Center in Dentistry "Dr. Keisaburo Miyata” School of Dentistry, Autonomous University of the State of Mexico, Toluca, MEX; 2 Academic Area of Dentistry, Institute of Health Sciences, Autonomous University of the State of Hidalgo, Pachuca, MEX

**Keywords:** ceramic brackets, co2 laser, orthodontic bond strength, orthodontics, phosphoric acid

## Abstract

Objective

This study evaluated the shear bond strength of ceramic brackets on enamel conditioned using three methods: CO_2_ laser, 37% phosphoric acid, and a combination of both, and analyzed the adhesive remnant index (ARI) to determine the amount of residual adhesive after bracket removal.

Material and methods

A total of 120 human premolars were stored in 0.2% thymol (Wt/vol) and divided into four groups (n=30/group): Group I was enamel conditioned with 37% phosphoric acid; Group II was irradiated with a CO_2_ laser at 0.5W power; Groups III and IV, 37% phosphoric acid; and CO_2_ laser was combined at powers of 0.5W and 3W, respectively. Brackets were bonded using Transbond Plus CC resin. Samples were light-cured for 15 seconds and stored (37°C, 24 h). ARI assessment: The amount of adhesive remaining on the enamel surface was determined using a scale of 0 to 3. The analysis of shear bond strength and ARI was performed using the Kruskal-Wallis Test.

Results

Statistical analysis revealed significant differences between some groups. Group III, which combined acid etching and CO_2_ laser conditioning, showed lower bond strength than the control group, although not significantly. On the other hand, Group II (CO_2_ laser only) showed the lowest bond strength and significant differences compared to the different groups, indicating that the exclusive use of laser is ineffective for conditioning. Significant differences were also observed between Groups III and IV, with Group IV exhibiting higher bond strength. However, the average value (GIII: 10.59±5.66 and GIV: 18.01±8.95 MPa) exceeded the recommended range (5.9 - 7.8 MPa). Regarding the ARI, Group II showed the lowest value, with significant differences compared to the other groups. Although Group IV had a higher ARI value than Group III, this difference was not statistically significant.

Conclusions

The combined use of phosphoric acid and CO_2_ laser provides adequate bond strength for ceramic brackets, with a lower amount of residual adhesive, which facilitates their removal and reduces the risk of damage to the enamel. Although laser irradiation alone does not provide sufficient bond strength, its use in combination with 37% phosphoric acid optimizes retention and minimizes the negative effects associated with conventional acid etching.

## Introduction

Dental enamel, predominantly composed of inorganic material, stands as one of the most resistant and organized tissues in the human body [1]. In the field of dentistry, research has been fundamental in developing techniques to optimize its adhesion with materials such as dental resins [2]. In 1955, Buonocore highlighted the effectiveness of acid etching, initially using 85% phosphoric acid, to improve enamel adhesion [3,4]. Subsequently, it was demonstrated that lower acid concentrations and shorter application times could also provide an optimal etching topography, leading to the use of 37% [5]. This technique, aimed at modifying the enamel surface to enhance adhesion, has been widely adopted in dentistry, leveraging phosphoric acid's ability to alter the interior of the prisms while maintaining the peripheral integrity of the enamel. However, its application is not without disadvantages, such as demineralization and color changes, which have driven the search for alternatives, including laser irradiation [6,7].

CO_2_ laser has emerged as a promising alternative for enamel etching. This method, by fusing and recrystallizing enamel prisms, creates a porous surface similar to the pattern produced by phosphoric acid. However, the lack of an ideal standard for CO_2_ laser enamel conditioning remains a challenge, highlighting the need for further research and experience in its application [8]. Since the 1970s, orthodontics has witnessed the evolution of next-generation adhesives, eliminating the need for individual bands on each tooth [9]. These adhesives mechanically incorporate into the irregularities of phosphoric acid-etched enamel, facilitating the bonding process between the enamel and the bracket base [10,11].

Ceramic brackets, valued for their aesthetics, have introduced new challenges in terms of conditioning and bonding protocols [12]. Their physical properties allow for greater light transmission during curing, leading to more effective polymerization of the adhesive, which can result in increased bond strength between the bracket and the tooth [13].

The present study addresses an important aspect that could strengthen the existing literature on enamel conditioning with CO_2_ laser and its combination with phosphoric acid. While there are advances in bonding techniques, this work has the potential to contribute in several key aspects. For example, limitations of previous laser conditioning methods, so far, previous studies have shown inconsistent results with the use of CO_2_ laser for enamel preparation, mainly due to variability in laser parameters (power, duration, distance) and their effect on bond strength. This study contributes by exploring controlled parameters and validating its effectiveness combined with phosphoric acid, something that few studies have examined [14-18]. Furthermore, there are inconsistencies in previous findings, as the literature shows discrepancies as to whether CO_2_ laser can be a viable or superior alternative to acid etching. The present study helps to resolve these inconsistencies by directly comparing different laser and acid combinations, providing data that may help improve the predictability of clinical outcomes [18-20]. Finally, by optimizing clinical protocols and evaluating not only bond strength but also adhesive remnant index (ARI), the study offers a practical clinical perspective on the ease of removal of ceramic brackets and enamel protection, something that benefits the orthodontic community in choosing better bonding methods.

In this study, we hypothesised that the combination of CO_2_ laser irradiation and 37% phosphoric acid etching would yield a significantly higher shear bond strength and less residual adhesive on tooth enamel following the removal of ceramic brackets, compared to the use of either phosphoric acid or CO_2_ laser alone. The objective of this study was to evaluate the shear bond strength of ceramic brackets on enamel conditioned using three methods: CO_2_ laser, 37% phosphoric acid, and a combination of both, and analyze the adhesive remnant index (ARI) to determine the amount of residual adhesive after bracket removal.

## Materials and methods

Design and sample

An in vitro experimental study was performed at the Advanced Studies and Research Center in Dentistry "Dr. Keisaburo Miyata," School of Dentistry, Autonomous University of State of Mexico, Toluca, Mexico. The selection of the sample size for this study was based on the review of previous research that evaluated similar situations using various types of lasers and enamel etching methods. For example, studies such as Kiryk et al. [21] used a reduced sample size (n=6 specimens per group), while Rajesh et al. [22] used a larger sample (n=30 specimens per group), demonstrating the variability in sample sizes in previous studies.

A power analysis was performed, a posteriori, using the G*Power program (Version 3.1.9.7, Heinrich-Heine-Universität Düsseldorf, Düsseldorf, Germany). The parameters entered into the analysis were: four groups, a large effect size (f=0.40), and an error of 0.05, with these parameters yielding a statistical power of 95.13% and an n of 112, which ensures a high probability of detecting significant differences between the groups, minimizing the risk of committing type II errors (false negatives). A power greater than 95% is generally considered more than adequate for experimental studies in dentistry, especially when a considerable effect size is expected. This approach allows to balance the statistical validity of the study with the practical feasibility of managing an adequate sample size in an experimental context, providing robust and clinically relevant results.

Tooth selection and sample preparation

A total of 120 human premolars extracted for orthodontic reasons were stored in 0.2% thymol (wt/vol). The buccal surface of each tooth was polished for 10 seconds using a low-speed rubber cup and fluoride-free prophylactic paste (ProphyTech, Zeyco Zapopan, Mexico). The teeth were then rinsed with water for 30 seconds and dried with oil-free compressed air.

Enamel conditioning

The teeth were randomly divided into four groups (n=30/group). Phosphoric acid at 37% (3M Unitek, California, USA) was used, along with CO_2_ laser irradiation (YOSHIDA Opelaser PRO, Tokyo, Japan) at a super pulse frequency.

**Figure 1:**
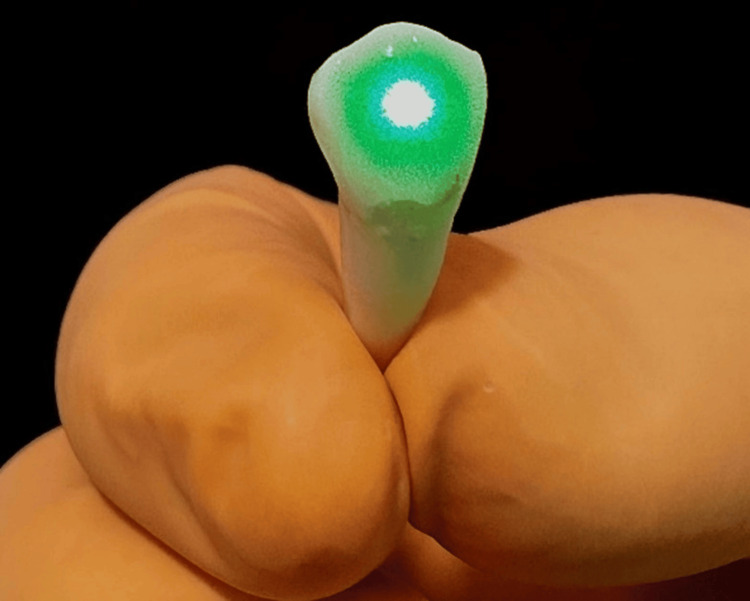
Application of CO2 laser irradiation on a tooth surface conditioned with 37% phosphoric acid

GI was conditioned with 37% phosphoric acid for 15 seconds. GII had CO_2_ laser applied at a distance of 5 cm, set to 0.5 W, for five seconds. GIII, GIV, both groups had 37% phosphoric acid applied to the surface. After three seconds, the CO_2_ laser was immediately applied at a distance of 5 cm for five seconds, with a power setting of 0.5W in GIII and 3W in GIV (Figure 1). After conditioning each group, the teeth were rinsed and dried with oil-free compressed air and water.

These specific power settings for the use of the CO_2_ laser in this study stem from the need to balance two key factors: achieving adequate bracket adhesion and minimizing the thermal impact on underlying dental tissues, especially the pulp. Previous studies have shown that the use of the CO_2_ laser in enamel conditioning can induce controlled changes (primarily related to ablation and surface chemistry modification of the enamel) in the tooth surface that improve bracket adhesion; therefore, precise control over the laser parameters is crucial to ensure the desired changes occur without compromising the tooth's health [23-26]. However, if the laser power is excessive, there is a risk of generating an increase in tooth temperature, which could affect the pulp irreversibly. Remembering that the effects of the laser on the target tissues will depend on the wavelength, output power, duration of exposure, and the amount of energy delivered to the tissue. Previous research has suggested that a temperature increase of more than 5.5°C in the pulp could lead to pulp necrosis. In this study, the CO_2_ laser parameters were carefully adjusted, with two power settings (0.5W and 3W) and a short exposure time (five seconds), based on previous studies that identified these values as safe to avoid overheating of the dental tissue. By using validated and moderate settings, as described in the literature, we aim to ensure that the power is sufficient to modify enamel without causing adverse thermal effects on the pulp [23,27-31].

Brackets

Ceramic brackets MBT prescription (0.022´´, Clarity Advanced, 3M Unitek, California, USA).

Bonding procedure

In all groups, the brackets were bonded using Transbond XT adhesive (3M Unitek, Monrovia, CA, USA). Transbond Plus Color Change resin (3M Unitek, California, USA) and light-cured with Ortholux (3M Unitek, California, USA) for 15 seconds.

A stainless steel wire (0.017 × 0.025´´) was placed in the slot of each bracket to prevent possible deformation during the debonding process. The teeth were embedded in acrylic resin using a template, ensuring that the buccal surface of each tooth was aligned parallel to the applied force during the shear bond strength test. They were then immersed in distilled water at 37°C for a period of 24 hours.

Shear bond strength

A load was applied in an occlusogingival direction at the bracket-tooth interface to generate a debonding force, using the AGS-X universal testing machine (Shimadzu Corp, Kyoto, Japan). Values were measured at a speed of 0.5 mm/min, and the data were recorded in Megapascals.

Adhesive remnant index (ARI)

After the shear bond strength test, the amount of residual adhesive on the tooth surface was evaluated using the original ARI scoring system [32]. The scale used was as follows: Score 0 = no adhesive left on the tooth; Score 1 = less than half of the adhesive left on the tooth; Score 2 = more than half of the adhesive left on the tooth; and Score 3 = all adhesive left on the tooth with a distinct impression of the bracket mesh.

The ARI assesses the amount of adhesive on the tooth after bracket removal. In clinical practice, a balance is sought between sufficient bond strength to hold brackets in place and minimizing adhesive residue to protect enamel integrity during debonding. Lower ARI scores are preferred because they mean minimal adhesive remains on the tooth after debonding. Adhesive removal becomes easier and reduces the risk of enamel damage, preventing demineralization and protecting tooth structure in the long term. The risk of bracket debonding increases if scores indicate weaker bond strengths. Bond failure occurs at the interface between the bracket and adhesive if ARI scores are high, suggesting excess adhesive remains on the enamel. Although it may indicate higher bond strength, this also means removing more adhesive after debonding, which can damage enamel if not done carefully [32].

Statistical analysis

Data were captured in Excel, and statistical analysis was performed in Stata (StataCorp LLC, College Station, TX, US). A univariate analysis was performed in which frequencies and percentages were reported for categorical variables and means and standard deviation for quantitative variables. One-way analysis of variance by ranks (Kruskal-Wallis Test) was used for bivariate tests; this is a non-parametric alternative to ANOVA when the data do not meet the assumptions of normality and homogeneity of variance required for conventional analysis of variance. This test is useful when more than two independent groups are compared and the distributions are not necessarily normal. This is especially relevant in biological or clinical experiments where samples may be small or where conditioning methods (such as the use of lasers or combinations of lasers and orthophosphoric acid) on enamel may produce results with non-normal distributions. A multiple comparison analysis was also performed, adjusting the p-value for four groups (the adjusted p-value for significance is 0.004167).

Ethical issues

The study protocol was reviewed and approved by the Research and Ethics Committee at the Autonomous University of the State of Mexico (UAEMex CEI-CIEAO-2023-03).

## Results

Shear bond strength

The descriptive results of the shear bond strength for each group are detailed in Table 1. Among the four groups, Group IV exhibited the highest mean shear bond strength value (18.01±8.95 MPa), indicating superior adhesive performance under the tested conditions. Conversely, Group II demonstrated the lowest mean strength (0.60±1.16 MPa), suggesting that the enamel conditioning method used in this group was not effective in achieving adequate bonding.

**Table 1:** Analysis of shear bond strength (MPa) Multiple comparisons between groups (Adjusted p-value for significance is 0.004167) S: statistically significant difference; NS: statistically NOT significant difference GI vs. GII: p=0.000000 (S); GI vs. GIII: p=0.033787 (NS); GI vs. GIV: p=0.175785 (NS); GII vs. GIII: p=0.000000 (S); GII vs. GIV: p=0.000000 (S); GIII vs. GIV: p=0.002895 (S)

Groups	Mean±SD
GI: Orthophosphoric acid 37% (control)	14.38±4.96
GII: CO_2_ laser (0.5W, 5 sec)	0.60±1.19
GIII: Orthophosphoric acid 37% + CO_2_ laser (0.5W, 5 sec)	10.59±5.66
GIV: Orthophosphoric acid 37% + CO_2_ laser (3W, 5 sec)	18.01±8.95

Statistical analysis using the Kruskal-Wallis test revealed significant differences between some groups (multiple comparison analysis, adjusted p-value for significance is 0.004167). Group III, which combined acid etching and CO_2_ laser conditioning (GIII: 10.59±5.66 MPa), showed lower bond strength compared to the control group but not significantly (GI: 14.38±4.9 MPa, adjusted p > 0.004), similar to what was observed in the comparison of GI vs. GIV (GIV: 18.01±8.95, p adjusted > 0.004) (Table 1). Meanwhile, Group II (CO_2_ laser only) displayed the weakest bonding performance overall, with statistically significant differences when compared to other groups (GI, GIII, and GIV, p adjusted < 0.004), underscoring the limitations of using CO_2_ laser alone as a conditioning method. Significant differences were also observed when comparing the values of GIII vs. GIV (p=0.002895), where GIV had higher shear bond strength values.

Adhesive remnant index

The descriptive results of the adhesive remnant index (ARI) values for each group are detailed in Table 2. Among the four groups, Group I (control) exhibited the highest mean amount of residual adhesive value (1.66±0.88), indicating superior adhesive performance under the tested conditions. Conversely, Group II demonstrated the lowest amount of residual adhesive (0.00±0.00), followed by GIII (1.06±0.63) and GIV (1.53±1.10).

**Table 2:** Frequency, percentage, and mean distribution of the adhesive remnant index (ARI) Multiple comparisons between groups (adjusted p-value for significance is 0.004167) S: statistically significant difference; NS: statistically NOT significant difference GI vs. GII: p=0.000000 (S); GI vs. GIII: p=0.025022 (NS); GI vs. GIV: p=0.244999 (NS); GII vs. GIII: p=0.000002 (S); GII vs. GIV: p=0.000000 (S); GIII vs. GIV: p=0.102170 (NS)

ARI scores (%)						
Group	(n)	0	1	2	3	Mean±SD
GI: Orthophosphoric acid 37% (control)	30	3 (10.0)	9 (30.0)	13 (47.3)	5 (16.7)	1.66±0.88
GII: CO_2_ laser (0.5W, 5 sec)	30	30 (100)	0	0	0	0.00±0.00
GIII: Orthophosphoric acid 37% + CO_2_ laser (0.5W, 5 sec)	30	4 (13.3)	21 (70.0)	4 (13.3)	1 (3.4)	1.06±0.63
GIV: Orthophosphoric acid 37% + CO_2_ laser (3W, 5 sec)	30	7 (23.3)	7 (23.3)	9 (30.1)	7 (23.3)	1.53±1.10

Statistical analysis using the Kruskal-Wallis test revealed significant differences between some groups (multiple comparison analysis, adjusted p-value for significance is 0.004167). Group I (control, orthophosphoric acid 37%) showed no statistically significant differences in adhesive remnant index with GIII (p=0.025022) and GIV (0.244999), which presented a lower ARI value but no significant difference (Table 1). Meanwhile, Group II (CO_2_ laser only) displayed the weakest ARI value, with statistically significant differences when compared to other groups (GI, GIII, and GIV, p adjusted < 0.004). Although the GIV group had a higher ARI value (1.53±1.10) than the GIII group (1.06±0.63), no statistically significant differences were observed (p=0.102170).

## Discussion

Shear bond strength

Effective enamel conditioning is critical for ensuring proper bracket adhesion, as it plays a decisive role in the stability and success of orthodontic treatment. The standard approach involves etching with 37% phosphoric acid, which selectively dissolves the superficial enamel layer, creating microporosities that facilitate the mechanical retention of dental attachments [33]. While this approach has proven effective in numerous clinical applications, it also presents significant drawbacks, including enamel demineralization and potential tooth discoloration following bracket removal [34].

Ceramic brackets emerged to meet the growing demand for more aesthetic orthodontic devices. They offer notable advantages, such as resistance to staining and discoloration, as well as chemical inertness to oral fluids, contributing to their durability [35]. However, they also present significant challenges due to their physical properties, which allow greater light transmission during bonding and create an excessive bond with adhesive resins [36]. Bond strength values have been established within the range of 5.9 to 7.8 MPa, a standard that remains relevant and safe in current clinical practice [37-39]. While this range offers some flexibility, an increase in bond strength may be associated with a higher risk of damage to dental tissue. For this reason, it is essential that the materials and bonding techniques used maintain these forces within safe limits to minimize complications during orthodontic treatment [36,37,40,41]. In response to these conditions, CO_2_ laser irradiation has been explored as an alternative for enamel conditioning. This technique avoids demineralization; however, studies have shown that its ability to provide adequate shear bond strength is variable and sometimes insufficient compared to phosphoric acid [42], mainly due to thermal effects on the tooth and its limited ability to withstand occlusal forces [2]. In this study, we evaluated three different conditioning methods: CO_2_ laser, 37% phosphoric acid, and combinations of both using varying acid exposure times and laser power settings. The laser irradiation parameters considered as reference values were tested and validated in previous projects to reduce the risk of side effects and increase the likelihood of positive outcomes [2,8,43,44].

The results obtained indicate that the bond strength required for ceramic brackets falls within an adequate range for clinical use when using the combination of laser and acid for conditioning, which is consistent with previous studies suggesting that the values obtained are sufficient to maintain the integrity of the adhesive bond without compromising dental enamel [44,45]. The discrepancies observed in bond strength between the different studies consulted could be due to the interaction of multiple factors, such as variability in laser parameters, conditioning methods used, adhesion measurement techniques, and experimental conditions. To address these differences, it would be valuable to perform comparative studies that standardize parameters and conditions or explore the variability of results in different clinical scenarios. In this regard, some hypotheses could be suggested for these differences. Regarding variability in laser parameters, differences in calibration and protocols used in the studies could explain the divergences in bond strength. For example, if a study uses a higher laser power than recommended, it could damage enamel and reduce the bonding capacity of orthodontic adhesive. Regarding the combination of conditioning methods, the combination of laser and acid could increase enamel roughness, improving micromechanical retention and, therefore, bond strength. In contrast, studies using only lasers may not achieve a surface texture suitable for optimal adhesion, which would explain the variability in results. Regarding the method of bond strength assessment, shear testing tends to yield more consistent and reproducible results, while tensile testing may be more sensitive to variations in enamel surface and adhesive type. This could explain why some studies report lower adhesion values. Regarding environmental conditions and artificial aging cycles, in in vitro studies, some investigations do not fully replicate real oral conditions, such as the presence of saliva, masticatory forces, temperature changes, and loading cycles. Some studies apply thermocycling or artificial aging cycles to simulate adhesive degradation over time, which may reduce bond strength and differ from studies that do not apply these cycles. Studies that do not consider factors such as thermocycling could overestimate bond strength, while those that do may offer more realistic results revealing a decrease in adhesion. Regarding the adhesives used, there are numerous types of orthodontic adhesives on the market, each with different chemical and physical properties. Therefore, the choice of adhesive could be crucial in obtaining optimal results. Some studies using adhesives less compatible with the roughness created by the laser could report lower values of adhesion strength. Finally, regarding enamel conditions, the intrinsic characteristics of enamel (e.g. thickness, quality, presence of hypomineralization, or dental fluorosis) may vary between studies. It is possible that some studies used teeth that already present enamel alterations, which would compromise the effectiveness of laser conditioning and, therefore, adhesion.

The use of CO_2_ laser in enamel conditioning presents several key advantages, particularly its ability to induce controlled changes in the dental surface, which promotes proper adhesion of ceramic brackets. The laser allows for precise modulation of the energy applied, significantly reducing the amount of residual adhesive after bracket removal and minimizing the risk of demineralization. Additionally, the reduction in application time optimizes procedural efficiency and decreases the risk of enamel overexposure. When combined with phosphoric acid, a conditioned surface is achieved for adhesion without compromising dental integrity. These characteristics make this technique an effective tool for improving the quality and safety of orthodontic treatments [23,43]. Statistical analysis revealed significant differences among the groups evaluated, specifically GIII treated with the combination of phosphoric acid and CO_2_ laser, compared to GII, which represents the standard for conditioning procedures. This finding is particularly relevant as it suggests that the combination of both methods could represent a significant improvement over conventional techniques, providing greater safety in terms of bracket retention without compromising dental structure.

Adhesive remnant index

In addition to debonding strength, we also evaluated the ARI to measure the amount of adhesive material remaining on the tooth surface after bracket removal, which has great clinical relevance, as less residual adhesive (0 and 1) is preferable to reduce cleanup work and minimize the risk of demineralization or enamel damage [46,47].

The findings indicated that Group III, treated with phosphoric acid and CO_2_ laser, had the lowest ARI, with 70% of samples showing a low index score. This suggests that this combination not only enhances initial adhesion but also eases bracket removal, minimizing adhesive residue on the enamel. Although the ARI offers valuable insights, it should be supplemented with other evaluation methods to fully understand the effects of bonding and debonding on dental enamel health. This study's in vitro conditions do not fully replicate the intraoral environment, where the bracket-enamel bond is influenced by saliva, biofilm, and mechanical load. A detailed analysis of the enamel surface after debonding, using techniques like scanning electron microscopy (SEM) [48], is needed, indicating the need for further research.

The present study presents limitations that need to be taken into account when interpreting the results properly. Although the sample size of 120 teeth (30 per group) is larger than the range observed in several studies, a larger sample size could increase internal validity and reduce the possibility of type II error (false negatives). Furthermore, variability in tooth type (e.g., premolars vs. other teeth) could affect the representativeness of the results. Furthermore, the research was in vitro, which limits the replicability of real conditions within the oral cavity. Saliva, oral microbiota, and biomechanical forces, among other factors, could influence bond strength in a real clinical setting. In vitro studies are useful for understanding basic mechanisms, but they do not fully capture the complexity of the oral environment, which limits the applicability of the results to clinical practice. Furthermore, the use of different laser settings and their impact on tooth temperature could vary between studies. Factors such as application distance, duration, and laser power may introduce technical variability that is not always uniformly controlled in each application. Bond strength was measured by shear strength testing, which is a standard method but is not the only approach to assess bond quality. Other methods, such as surface wear assessment or scanning electron microscopy (SEM) studies, could have provided additional information on morphological changes in enamel.

## Conclusions

The combined use of phosphoric acid and CO_2_ laser (GIII) provides adequate bond strength for ceramic brackets with a lower amount of residual adhesive, facilitating their removal and reducing the risk of damage to enamel. Although laser irradiation alone does not provide sufficient bond strength, its use in combination with acid optimizes retention and minimizes the negative effects associated with conventional acid etching. It is recommended to explore its application in different types of brackets and clinical conditions to validate these findings.
